# The Effect of Polyacrylate Emulsion Coating with Unmodified and Modified Nano-TiO_2_ on Weathering Resistance of Untreated and Heat-Treated Wood

**DOI:** 10.3390/polym16040511

**Published:** 2024-02-14

**Authors:** Josip Miklečić, Martina Zeljko, Sanja Lučić Blagojević, Vlatka Jirouš-Rajković

**Affiliations:** 1 Department of Wood Technology, Faculty of Forestry and Wood Technology, University of Zagreb, 10000 Zagreb, Croatia; vjirous@sumfak.unizg.hr; 2 M PLAST d.o.o., 88220 Široki Brijeg, Bosnia and Herzegovina; martinazeljko7@gmail.com; 3 Department of Surface Engineering of Polymer Materials, Faculty of Chemical Engineering and Technology, University of Zagreb, 10000 Zagreb, Croatia; slucic@fkit.unizg.hr

**Keywords:** weathering, nano-TiO_2_, heat-treated wood, coating

## Abstract

In this research, the influence of titanium dioxide (TiO_2_) nanoparticles and their modifications on the weathering resistance of untreated and heat-treated wood was studied. The wood samples were coated with polyacrylate waterborne emulsion coatings that contain nano-TiO_2_ in the amount of 0.75 wt.%. Two types of modifiers were used to modify the nano-TiO_2_ surface: 2,2′-azobis(2-methylpropionamide) dihydrochloride (AIBA) and 3-aminopropyltrimethoxy silane (AMPTS). Coated and uncoated wood samples were exposed to accelerated weathering by application of sunlight, water and moisture for 360 h. During the research, the dry film thickness, color, gloss and hardness of the surface of the samples were measured. The obtained results showed that the effect of the addition and surface modification of nano-TiO_2_ on the color and gloss stability was different on untreated and heat-treated ash wood, and that accelerated weathering causes an increase in surface hardness and a decrease in thickness of the dry coating.

## 1. Introduction

In outdoor use, wood is subject to slow surface degradation known as weathering. Many environmental factors such as sunlight, moisture, temperature and atmospheric gases affect the weathering. It is known that the UV component of sunlight is the most important factor responsible for depolymerization of lignin in cell walls, followed by leaching of degradation products, causing surface erosion [1,2]. Changes in appearance (color and gloss), surface roughness and surface checking that are the result of weathering significantly reduce the aesthetic value and performance of wood as a material for outdoor use. Although heat-treated wood has improved properties compared to unmodified wood, such as increased dimensional stability and better water repellency, it is also susceptible to weathering [3] with a slightly slower degradation rate than unmodified wood [4,5]. In order to protect wood from weathering and maintain the original appearance of wood, the most preferable method is to use clear coatings as protection. Nowadays, waterborne coatings are preferred due to increasingly strict environmental regulations and user (consumer) awareness of climate and environmental issues. Waterborne acrylic polymers are often used as wood-coating materials because of their good water resistance, film clarity and UV resistance [6,7]. Clear coatings have the advantage of highlighting the beauty of the wooden surface, but they are sensitive to solar radiation, which can degrade the film and decompose the wood underneath. It has been shown that coating wood with a clear waterborne acrylic coating is ineffective against photodegradation [8] and that it is necessary to add a UV absorber as additive in the coating that will prevent the transmittance of UV light into the wood. In recent years, nano-titanium dioxide (TiO_2_) has attracted interest as an inorganic UV absorber [9,10,11]. Titanium dioxide has good ultraviolet (UV)-blocking power and some attractive properties, such as long-term life time and thermal stability compared to standard organic UV-absorbers [12]. Titanium dioxide nanoparticles in the crystal form of rutile, compared to organic UV absorbers, offer protection from longer wavelengths above 400 nm, by light scattering and, below 400 nm, by UV-light absorption [13]. Moreover, they do not degrade and migrate during weathering. However, TiO_2_ nanoparticles may also exhibit photocatalytic behavior by formation of reactive free radicals when absorbing UV-rays, which can degrade the surrounding material [14,15]. Moreover, they also have a high tendency of agglomeration, which can affect the properties of polymeric nanocomposite materials [16]. It has been established that surface modification of TiO_2_ with silica [17,18] or alumina (Al_2_O_3_) [19] can suppress the photocatalytic property of TiO_2_. The surface modification of TiO_2_ nanoparticles with silane coupling agents [12,20,21,22,23,24] and titanate coupling agents [25] was shown to improve the dispersibility of TiO_2_ nanoparticles in an acrylic emulsion coating. Zeljko et al. [26] investigated the effect of surface modification of TiO_2_ on the properties of polyacrylate coatings for UV protection, comparing four different modifiers: nonionic emulsifier TX-100, the cationic initiator AIBA, the silane AMPTS, and the trisilanol POSS. It has been shown that the modification of a TiO_2_ surface with silane AMPTS increased the UV-absorbing ability of TiO_2_, while modification with other modifiers increased the photocatalytic effect of TiO_2_. Ash wood was chosen because it is an autochthonous species in Croatia and following heat treatment at high temperature without the presence of air exhibits enhanced stability and resistance to rot and decay, making it a suitable choice for exterior applications, such as decking, siding, fencing, and outdoor furniture. Due to its enhanced durability and stability, heat-treated ash wood can be used in place of more expensive tropical wood species and is an environmentally responsible option. However, like standard wood, heat-treated wood is also susceptible to weathering induced by ultraviolet radiation from sunlight, which significantly reduces wood’s aesthetic values and performance. Therefore, finding a coating that protects the wood surface from photochemical degradation during weathering is important for extending the life of the wood product. The aim of this study was to investigate the effectiveness of clear polyacrylate waterborne emulsion coatings with added unmodified or modified TiO_2_ nanoparticles in protecting untreated and heat-treated ash wood from photodegradation during accelerated laboratory exposure. This was assessed by measuring color, gloss, dry film thickness and hardness changes and by macroscopic evaluation of the coated samples.

## 2. Materials and Methods

In this study, samples of untreated and heat-treated ash wood, with a radial texture and without visible defects, were used. Wood samples were commercially thermally modified using the ThermoWood^®^ process with a peak temperature of 190 °C without the presence of air. The dimensions of the samples were 130 mm × 45 mm × 10 mm (L × R × D). Prepared wood samples were conditioned at (23 ± 2) °C and (50 ± 5)% relative humidity to the constant mass and then coated with polyacrylate waterborne emulsion coatings with the addition of titanium dioxide (TiO_2_) nanoparticles. Emulsion coatings were prepared by in situ emulsion polymerization with a nano-TiO_2_ content of 0.75 wt.%. The materials used in emulsion polymerization are shown in Table 1. TiO_2_ nanoparticles were surface-modified with two types of modifiers: 2,2′-azobis(2-methylpropionamide) dihydrochloride (AIBA) and 3-aminopropyl)trimethoxy silane (AMPTS). For testing, four types of polyacrylate waterborne emulsion coatings were prepared (Table 2), the preparation procedure of which is described in the paper by Zeljko et al. [26].

The surface of the wood samples was manually sanded with P150 grit sandpaper before applying the emulsion coatings. Three untreated and three heat-treated wood samples were prepared for each type of emulsion coating. The emulsion coatings were applied by hand with a brush in three layers in the amount of 120 g/m^2^ per layer. Each layer of emulsion coating was dried for 24 h at room conditions. After drying, the first and second layers were sanded by hand with P240 grit sandpaper before applying the next layer. In addition to the coated wood samples, three uncoated untreated and three uncoated heat-treated wood samples were prepared, which were only manually sanded. The ends of all wood samples were sealed with two layers of two-component epoxide coating to prevent water absorption through the ends of the wood samples.

The prepared samples were exposed to accelerated weathering, i.e., to sunlight, water and moisture in a Q-SUN XE-2 device (Q-Lab, Bolton, UK) equipped with a xenon lamp and a daylight filter (Daylight-Q). The exposure of the samples lasted a total of 360 h under the conditions according to EN ISO 16474-2:2013 [27] (test method A) shown in Table 3. During the exposure, the samples rotated around the xenon lamp.

The color of the surface of the samples was measured with an xRite Ci64 spectrophotometer (X-Rite, Grand Rapids, MI, USA) at five measuring points per sample before weathering and after 24, 48, 72, 96, 120, 240 and 360 h of weathering. During the measurement, the following settings of the spectrophotometer were used: measuring geometry d/8°, standard observer 10°, light source D65 and measuring-aperture diameter 8 mm. To determine the total color change (ΔE*), the CIE L*a*b* color system was used, in which L* denotes brightness, while a* and b* are chromaticity coordinates on the red-green and yellow-blue axis, respectively. The color change was calculated according to the following formula $∆E^{*}=\sqrt{{\left({∆L}^{*}\right)}^{2}+{\left({∆a}^{*}\right)}^{2}+{\left({∆b}^{*}\right)}^{2}}$ where ΔL* = L*after exposure—L*before exposure, Δa* = a*after exposure—a*before exposure, Δb* = b*after exposure—b*before exposure.

The surface gloss of the samples is measured in the same way as the color before weathering and after 24, 48, 72, 96, 120, 240 and 360 h of weathering. The gloss was measured with a KSJ reflectometer at an angle of 60° in the direction of the grain at three measuring points per sample.

The hardness of the coated surface of the samples was measured before and after 360 h of accelerated weathering with a König’s pendulum at three measuring points per sample. The time of pendulum damping was measured from 6° to 3°, where a longer damping time indicated that the coated surface was harder. König’s pendulum was calibrated using an uncoated glass plate 5 mm thick, so that the damping time was 250 ± 10 s.

The dry film thickness and the surface of the emulsion coatings were examined before and after accelerated weathering using a ZEISS AXIO ZOOM V16 optical microscope (Carl Zeiss AG, Oberkochen, Germany) at a magnification of 20x on the cross-section of the samples that were gradually finely sanded with P1500 final paper grit size.

For a visual assessment of the degradation of the wood surface, the samples were regularly scanned using a desk top scanner at a resolution of 300 DPI resolution (Canon 2520 MFP, Canon, Tokyo, Japan) before and during accelerated weathering.

## 3. Results and Discussion

### 3.1. Color Change

By finishing untreated and heat-treated ash wood with the PA emulsion coating, the color of the surface of the samples changed even though the PA emulsion coatings were transparent (Table 4). A greater color change was observed on the dark heat-treated samples than on the light untreated ash samples. Although the addition of nano-TiO_2_ and the modification of nano-TiO_2_ reduced the transmittance of the PA emulsion coating in the visible part of the spectrum [26], only the modification of nano-TiO_2_ with AIBA caused an intense color change of the heat-treated ash surface (more than twice) compared to the PA emulsion coating without nano-TiO_2_. With AIBA modification of nano-TiO_2_, a milky coloration of the surface of heat-treated ash wood is visible. This effect is associated with the agglomeration of nano-TiO_2_ modified with AIBA [26].

**Figure 1:**
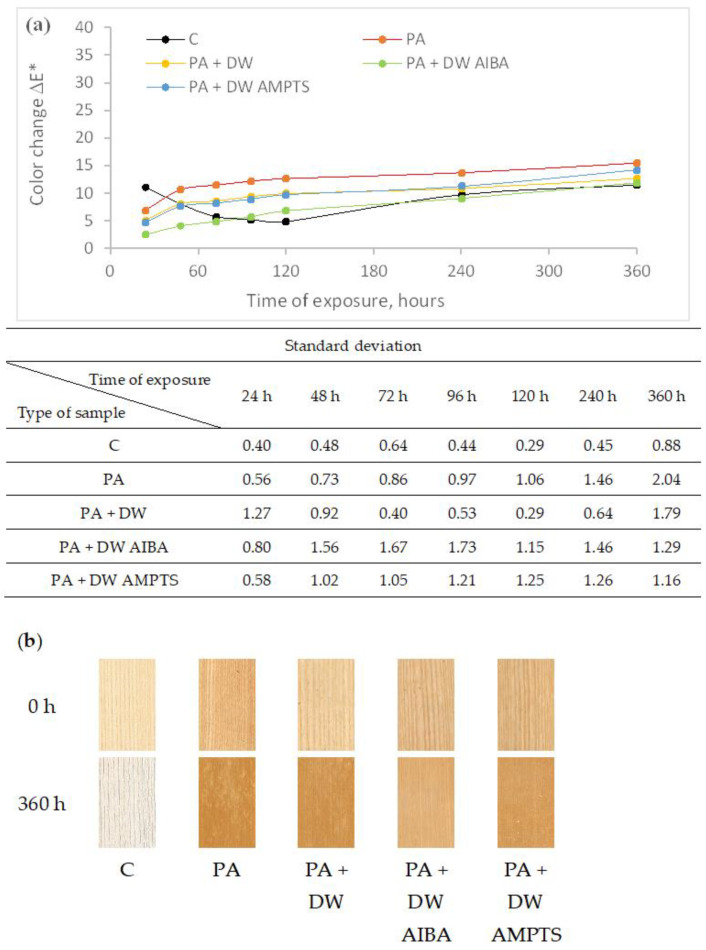
Color change of untreated ash wood samples during artificial weathering (**a**) and images of unexposed and 360 h weathered ash wood samples (**b**) (C—uncoated, PA—emulsion without nanoparticles, PA + DW—emulsions with 0.75% nano-TiO_2_ without modification, PA + DW AIBA—emulsions with 0.75% nano-TiO_2_ modified with AIBA, PA + DW AMPTS—emulsions with 0.75% nano-TiO_2_ modified with AMPTS).

Figure 1a clearly shows the different trend in color change of uncoated and coated untreated ash wood samples. That is the consequence of intensive darkening of the surface of the uncoated untreated samples at the beginning of the accelerated weathering, after which it starts to lighten, while the surface of the coated untreated samples gradually becomes lighter during the accelerated weathering. The addition of unmodified and modified nano-TiO_2_ into the PA emulsion coating increases the color stability of coated unmodified samples. At the end of accelerated weathering, the color change of the surface finished with PA emulsion coatings with nano-TiO_2_ was smaller compared to the surface finished with PA emulsion coatings without nano-TiO_2_ by 18, 24 and 8% for PA + DW, PA + DW AIBA and PA + DW AMPTS emulsion coatings, respectively. The influence of nano-TiO_2_ modification on the stability of the surface color was greater at the beginning than at the end of the accelerated weathering. Although the color change values of the uncoated unmodified samples were less than the color change values of the coated unmodified samples (Figure 1a), the color change effect visible to the naked eye was more pronounced on the uncoated than on the coated samples (Figure 1b) due to the intense change in lightness of uncoated samples.

**Figure 2:**
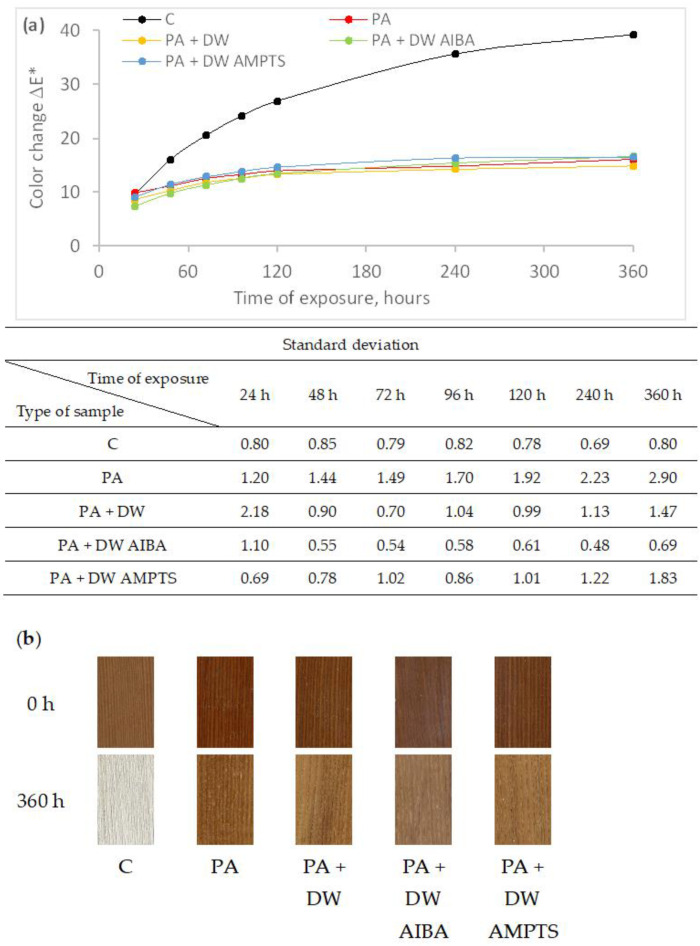
Color change of heat-treated ash wood samples during artificial weathering (**a**) and images of unexposed and 360 h weathered ash wood samples (**b**) (C—uncoated, PA—emulsion without nanoparticles, PA + DW—emulsions with 0.75% nano-TiO_2_ without modification, PA + DW AIBA—emulsions with 0.75% nano-TiO_2_ modified with AIBA, PA + DW AMPTS—emulsions with 0.75% nano-TiO_2_ modified with AMPTS).

In contrast to untreated ash wood samples, the difference in color change between uncoated and coated heat-treated ash wood samples was highly pronounced (Figure 2) and all samples became lighter during accelerated weathering. The PA emulsion coating significantly reduced the color change of heat-treated ash wood, but the addition of nano-TiO_2_ and modification of nano-TiO_2_ did not increase the color stability of the surface coated with the PA emulsion coatings.

### 3.2. Gloss Change

**Figure 3:**
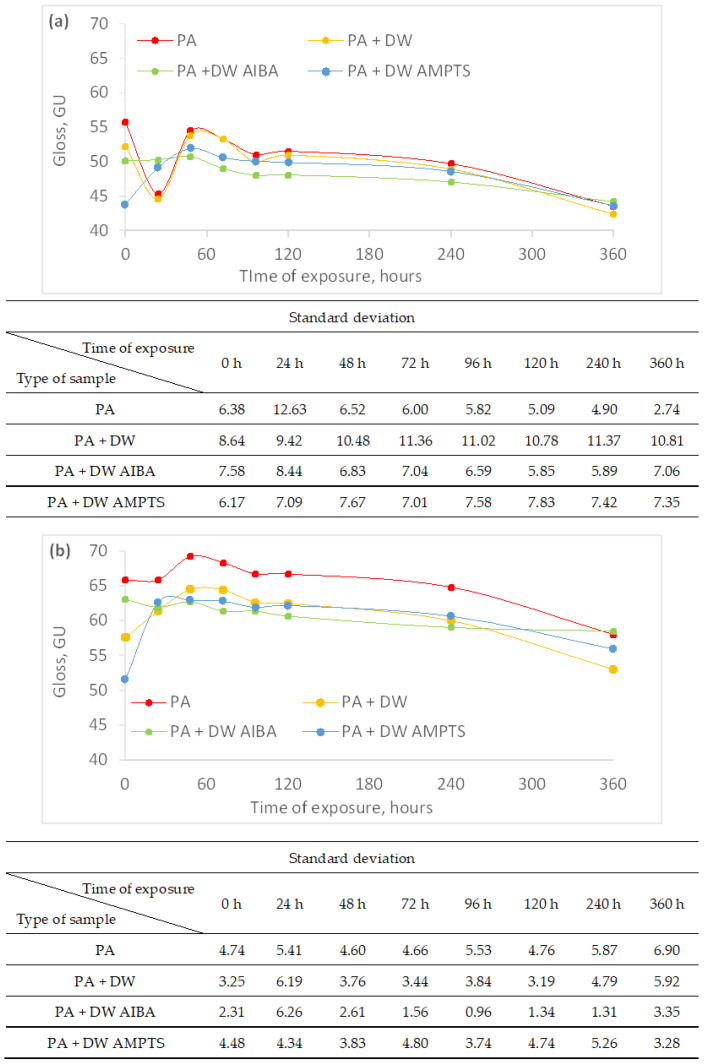
Gloss of surface of untreated (**a**) and heat-treated (**b**) ash wood samples during artificial weathering (C—uncoated, PA—emulsion without nanoparticles, PA + DW—emulsions with 0.75% nano-TiO_2_ without modification, PA + DW AIBA—emulsions with 0.75% nano-TiO_2_ modified with AIBA, PA + DW AMPTS—emulsions with 0.75% nano-TiO_2_ modified with AMPTS).

By coating the wood samples, the gloss of the surface increased significantly, which was expected. However, the addition of nanoparticles reduced the initial gloss of all emulsion coatings (Figure 3). The same result was obtained by Pazokifard et al. [28], who attributed the reduction in coating gloss to the formation of nano-TiO_2_ agglomerates on the surface of the coating. The initial surface gloss values of the coated samples were higher on heat-treated ash wood (Figure 3b) by 17% on average compared to untreated ash wood (Figure 3a). Furthermore, accelerated weathering caused a surface gloss reduction in coated untreated and heat-treated ash wood (Figure 3). The addition of nano-TiO_2_ increased the gloss stability of PA-coated untreated and heat-treated ash wood during accelerated weathering, which was also determined by Pazokifard et al. [28]. However, as with the color change, modification of nano-TiO_2_ did not increase the gloss stability of PA-coated heat-treated wood, compared to unmodified nano-TiO_2_. In addition, at the end of accelerated weathering, the gloss change of the surface finished with PA emulsion coatings with nano-TiO_2_ was smaller compared to the surface finished with net PA emulsion coating by 19, 51 and 98% on unmodified samples and by 42, 43 and 44% on heat-treated samples for PA + DW, PA + DW AIBA and PA + DW AMPTS emulsion coating, respectively. Based on the increase in gloss stability, it can be concluded that nano-TiO_2_ increased the performance of the PA coating, which is also stated by Weldon [29].

### 3.3. Surface Hardness and Dry Film Thickness

**Figure 4:**
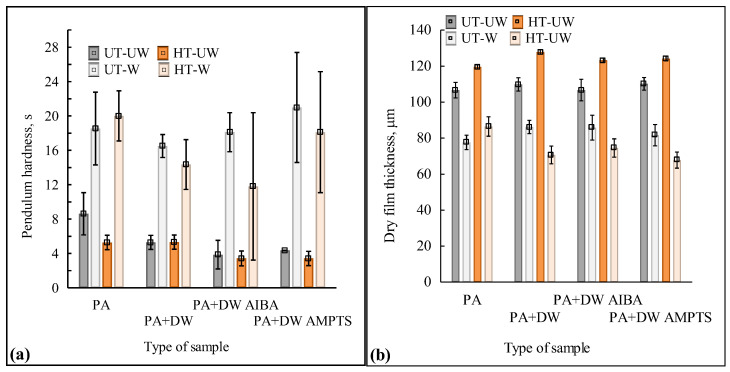
Surface hardness (**a**) and dry film thickness (**b**) of coated untreated (UT) and heat-treated (HT) ash wood samples before (UW) and after (W) accelerated weathering. Vertical bars denote standard deviation. (PA—emulsion without nanoparticles, PA + DW—emulsions with 0.75% nano-TiO_2_ without modification, PA + DW AIBA—emulsions with 0.75% nano-TiO_2_ modified with AIBA, PA + DW AMPTS—emulsions with 0.75% nano-TiO_2_ modified with AMPTS).

**Figure 5:**
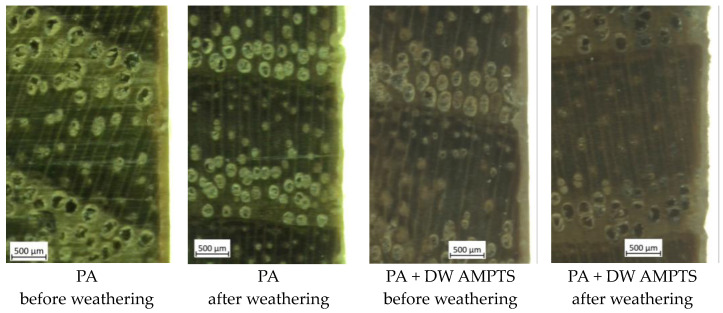
Micrographs (magnification 10.5×) of a cross-section of heat-treated ash wood finished with net PA and PA + DW AMPTS emulsion coating showing the surface of the coating before and after accelerated weathering.

The results of surface hardness in Figure 4a show that accelerated weathering increased the surface hardness of coated untreated and heat-treated ash wood samples. A number of authors also found an increase in the surface hardness of wood coated with an acrylic water-based coating after weathering [30,31,32]. The obtained increase in the hardness of the coated wood surface under the influence of accelerated weathering can be related to structural changes and crosslinking in coatings caused by UV radiation, which was determined for free films of emulsion coatings [26]. However, the decrease in film thickness after accelerated weathering (Figure 4b) can also affect the increase in surface hardness, because Ma et al. [33] found that the pendulum hardness of organic coatings is greatly influenced by coating thickness. Furthermore, no pronounced differences were found in surface hardness between emulsion coatings with and without nano-TiO_2_ and between unmodified and modified nano-TiO_2_. After accelerated weathering, the thickness of the coating film decreased (Figure 4b) on average by 24% on untreated and by 39% on heat-treated ash wood samples. Hu et al. [34] state that weathering causes gradual erosion of the surface of organic coatings, while erosion of the coating causes a significant reduction in coating thickness and leads to loss of gloss and color change [35], which coincides with the results shown in Figure 1, Figure 2 and Figure 3. Despite the reduced thickness of the film, a crack in the coating was not observed with a microscope regardless of heat treatment of the wood and type of coating, but the surface of the coating became uneven, as can be seen in Figure 5. The surface hardness values are relatively low, which indicates that the emulsion coatings have high elasticity, so even with increased hardness and reduced film thickness, their elasticity did not change to a large extent.

## 4. Conclusions

Net PA emulsion coating and the addition of nano-TiO_2_ had a different effect on color stability during accelerated weathering on untreated and heat-treated ash wood. Nano-TiO_2_ and AIBA surface modification of nano-TiO_2_ increased the color stability of PA emulsion coating on untreated ash wood. However, on heat-treated ash wood, the net PA emulsion coating significantly reduced the color change during accelerated weathering, and the addition and surface modification of nano-TiO_2_ did not further increase the color stability of PA emulsion coating. Furthermore, nano-TiO_2_ increased the gloss stability of PA emulsion coating on untreated and heat-treated ash wood. In addition, surface modification of nano-TiO_2_ increased the gloss stability of PA emulsion coating on untreated ash wood, but had no effect on the gloss stability on heat-treated ash wood. Accelerated weathering increased the surface hardness and decreased the dry film thickness of PA emulsion coating on untreated and heat-treated ash wood, regardless of the addition and surface modification of nano-TiO_2_.

## Figures and Tables

**Table 1:** Materials used in emulsion polymerization.

Monomers	Methyl methacrylate Butyl acrylate
Nano-filler	Titanium dioxide (average particle size of 30 nm)
Dispersion medium	Demineralized water
Emulsifier	Disponil FES 77 (Sodium dodecyl sulfate)
Initiator	Ammonium persulfate

**Table 2:** Prepared water-borne emulsion coatings.

Type of Emulsion Coating	Mark
Without nanoparticles	PA
With 0.75% nano-TiO_2_ without modification	PA + DW
With 0.75% nano-TiO_2_ modified with AIBA	PA + DW AIBA
With 0.75% nano-TiO_2_ modified with AMPTS	PA + DW AMPTS

**Table 3:** Conditions during accelerated weathering.

Exposure Period	Irradiance at 340 nm W/(m^2^⋅nm)	Black-Standard Temperature °C	Chamber Temperature °C	Relative Humidity %
102 min dry	0.51 ± 0.02	65 ± 3	38 ± 3	50 ± 10
18 min water spray	0.51 ± 0.02	-	-	-

**Table 4:** Color change of coated untreated and heat-treated ash wood samples after applying PA emulsion coatings (standard deviations are shown in parentheses).

Type of Emulsion	Untreated Ash Wood				Heat-Treated Ash Wood			
	ΔE*	ΔL*	Δa*	Δb*	ΔE*	ΔL*	Δa*	Δb*
PA	7.0 (1.30)	−4.1 (1.13)	2.9 (1.42)	4.8 (0.25)	8.3 (0.71)	−7.8 (0.87)	2.5 (0.24)	0.3 (1.17)
PA + DW	5.0 (0.47)	−2.4 (0.30)	1.5 (0.25)	4.1 (0.40)	7.0 (0.74)	−6.2 (0.62)	1.9 (0.31)	−2.6 (1.22)
PA + DW AIBA	3.6 (0.99)	−1.3 (0.35)	1.6 (0.21)	−2.9 (1.16)	18.5 (1.73)	−1.4 (0.80)	−0.8 (0.48)	−18.5 (1.75)
PA + DW AMPTS	4.3 (0.50)	−3.2 (0.50)	2.6 (0.18)	0.7 (0.58)	8.7 (1.09)	−5.5 (0.82)	1.3 (0.41)	−6.6 (1.35)

## Data Availability

The raw data supporting the conclusions of this article will be made available by the corresponding author on request due to privacy.
